# NK and NKT cells in the diagnosis of diffuse lung diseases presenting with a lymphocytic alveolitis

**DOI:** 10.1186/s12890-019-0802-1

**Published:** 2019-02-13

**Authors:** Oksana Sokhatska, Eva Padrão, Bernardo Sousa-Pinto, Marília Beltrão, Patrícia Caetano Mota, Natália Melo, Luís Delgado, António Morais

**Affiliations:** 1 Basic & Clinical Immunology Unit, Department of Pathology, Faculty of Medicine, Alameda Professor Hernâni Monteiro, University of Porto, 4200-319 Porto, Portugal; 2 CINTESIS - Center for Health Technology and Services Research, Faculty of Medicine, University of Porto, Porto, Portugal; 3 Department of Pulmonology, Hospital de São João and Faculty of Medicine, University of Porto, Porto, Portugal; 4 MEDCIDS – Department of Community Medicine, Information and Health Decision Sciences, Faculty of Medicine, University of Porto, Porto, Portugal

**Keywords:** Bronchoalveolar lavage fluid, Diffuse lung diseases, Hypersensitivity pneumonitis, NKT cells, Natural killer cells, Sarcoidosis

## Abstract

**Background:**

Diffuse lung diseases (DLD) are characterized by different immunophenotypes in the bronchoalveolar lavage fluid (BALF). We aimed to evaluate the diagnostic value of BALF NK and NKT cell counts of patients with DLD and lymphocytic alveolitis.

**Methods:**

We assessed 202 patients with DLD, who underwent BALF immunophenotyping. Samples were routinely processed by flow cytometry and lymphocyte subsets were compared between patients with sarcoidosis (*n* = 106), hypersensitivity pneumonitis (HP; *n* = 53), and other DLDs (*n* = 43). We compared absolute counts and percentages of NK and NKT cells between patients with HP versus the remaining DLD patients. To assess the accuracy of BALF lymphocyte subsets in the diagnosis of HP, we calculated the respective areas under the receiver operating characteristic curves (AUC-ROC).

**Results:**

Patients with HP had significantly higher numbers of BALF NK cells, and its percentage was significantly associated with a higher odds of HP, even after adjustment for the NKT and CD8^+^ cells. For the absolute number of BALF NK cells, we found an AUC-ROC of 0.76 (95%CI = 0.68–0.84) when comparing patients with HP versus the remaining DLD. The cut-offs of 2000 NK cells/mL and of 2.4% NK cells in the BALF had a specificity and a negative predictive value over 80% for the diagnosis of HP. BALF NK cells absolute counts were significantly higher in HP patients with a restrictive pattern. No such differences were observed for NKT cells.

**Conclusions:**

BALF NK immunophenotyping may be a helpful adjunct to the diagnostic work-up of DLD, particularly in the differential diagnosis of HP.

**Electronic supplementary material:**

The online version of this article (10.1186/s12890-019-0802-1) contains supplementary material, which is available to authorized users.

## Background

Diffuse lung diseases (DLD) are a heterogeneous group of disorders, many of them with an unknown etiology, that are characterized by interstitial lung immune/inflammatory cell infiltrates of variable intensity and composition [1, 2]. A multidisciplinary approach is essential for establishing an accurate diagnosis. Analysis of bronchoalveolar lavage fluid (BALF) from the interstitial inflammation is a standardized procedure that is widely available within the diagnostic work up of patients with DLD [2, 3]. Although certain BALF cell profiles have been linked to specific DLDs, they cannot provide a definitive diagnosis [4, 5]. In addition, the diagnostic value of certain cell immunophenotypes, including natural killer (NK) and NKT cells (CD3^+^ 16/56^+^ cells), needs to be further evaluated [6–9].

NK cells are innate lymphocytes with a cytotoxic function and an ability to secrete several pro- and anti-inflammatory cytokines [10]. NKT cells, in turn, express features of both NK and T cells, and are able to rapidly produce cytokines, modulate the T_H_1/T_H_2 balance, and stimulate, or on the contrary, suppress immune responses [11–13]. There have been recent reports that patients with hypersensitivity pneumonitis (HP) have a higher BALF NKT cells percentage than patients with sarcoidosis and healthy controls [6, 7, 9]. Higher NK and NKT-like cell counts have also been detected in the BALF of patients with organizing pneumonia (OP) compared with those with idiopathic pulmonary fibrosis (IPF) or healthy controls [14, 15]. Deficient or impaired NKT cells function may also have a role in the pathogenesis of sarcoidosis (reviewed in [11]). In brief, although the exact role of BALF NK and NKT cells in DLD has not been fully established, assessment of these cell subtypes by flow cytometry may be a useful supplement for the diagnosis of DLD, particularly as these cells in the lungs have immune regulatory functions and may be involved in the modulation of the bronchoalveolar inflammation [16]. Therefore, the aim of this study was to characterize and compare, using flow cytometry immunophenotyping, BALF NK and NKT cell populations in patients with DLD and lymphocytic alveolitis, evaluating how they relate to their clinical presentations.

## Methods

### Study population

We prospectively included a total of 202 patients observed at the DLD Outpatient Clinic at Centro Hospitalar de São João, a tertiary referral public hospital in Porto, northern Portugal. All patients having lymphocytic alveolitis (> 15% BALF lymphocytes) detected by BALF differential cell counts within the diagnostic workup of a suspected diffuse lung disease were included; patients with < 15% lymphocytes in BALF were excluded from the analysis. They were assessed using a multidisciplinary diagnostic approach combining clinical, radiologic, and BALF findings (total and differential cell counts and CD4/CD8 ratio) and, in some cases, histopathologic evaluation according to the American Thoracic Society/European Respiratory Society statements [17–20]. The patients were subsequently categorized into 3 groups: a sarcoidosis group, an HP group and an other DLD group. All the patients in the sarcoidosis group fulfilled the European Respiratory Society/American Thoracic Society/World Association of Sarcoidosis and Other Granulomatous Diseases statement on sarcoidosis, and thoracic involvement was classified according to the Scadding criteria [19, 21]. HP was diagnosed using the criteria proposed by Lacasse et al. [22]. In the other DLD group, (i) IPF was diagnosed according to the European Respiratory Society/American Thoracic Society/Japanese Respiratory Society/Latin American Thoracic Society Guidelines; (ii) connective tissue disease with pulmonary involvement (CTD-ILD) diagnosis was based on high-resolution computed tomography (HRCT) findings, BALF features, and a previous CTD diagnosis; and (iii) OP was diagnosed by histologic examination of lung samples obtained by CT-transthoracic lung biopsy [17, 20, 23–25].

### Bronchoalveolar lavage fluid and flow cytometry

BALF was obtained within the diagnostic approach of patients on an outpatient basis, in accordance with the technical recommendations of the European Respiratory Society Task Group on BALF [26]. Briefly, the lavage was performed in the subsegmental bronchus of the middle lobe or lingula using 4 × 50 mL saline pre-warmed to 37ºC, with gentle aspiration after each instillation. The last three recovered samples were homogenized and analyzed for total cellular counts (Neubauer chamber) and viability (trypan blue exclusion) determined. A total of 500 cells were counted on Wright-Giemsa stained cytospin slides. The samples were processed by routine flow cytometry analysis in our laboratory with the following monoclonal antibodies combinations in two different tubes - tube 1 contained a combination of anti-CD16-PE (Clone 3G8) (BD Pharmingen, San Diego, CA, USA), anti-CD3-FITC (Clone SK7), anti-CD56-Pe (CloneMY31), anti-CD45-PerCP-Cy5.5 (Clone2D1), and anti-CD19-APC (Clone SJ25C1); tube 2 was prepared with anti-CD4-FITC (Clone SK3), anti-CD8-Pe (Clone SK1), anti-CD45-PerCP-Cy5.5 (Clone2D1), anti-CD3-APC (CloneSK7) (all BD Biosciences, San Jose, CA, USA). The samples were run through a BD FACS Calibur™ flow cytometer (BD Biosciences, San Jose, CA, USA) and analyzed using BD CellQuest software (BD Biosciences, San Jose, CA, USA), with the acquisition of a minimum of 10,000 total events [27–29]. Lymphocytes were distinguished on the basis of forward (FSC) versus side (SSC) scatters and additional gating was applied using SSC versus CD45 to distinguish between leukocyte populations (namely lymphocytes) and cell debris. Subsequently, we applied SSC versus CD3, CD19 and CD16/56, in order to respectively identify T lymphocytes, B lymphocytes, and NK cells. T lymphocytes were gated based on SSC versus CD3, with T lymphocytes subpopulations being identified as CD3^+^CD4^+^ (T-helper) and CD3^+^CD8^+^ (T-cytotoxic) cells. B lymphocytes were gated based on SSC versus CD19. Finally, we applied CD3 versus CD16/56 markers to identify NK (CD3^−^ 16/56^+^ cells) and NKT cells (CD3^+^ 16/56^+^) (gating strategy depicted in Additional file 1). All aforementioned cell populations were scored as percentages of lymphocytes. Lymphocyte subpopulations in a parallel peripheral blood sample were analyzed in a similar fashion.

### Statistical analysis

Results are presented as means and standard deviations (SD) or as medians and quartiles (percentile 25 and percentile 75) for continuous variables, and as absolute frequencies and proportions for categorical variables. The Mann-Whitney U-test, Kruskal-Wallis test and Chi-squared test were used as appropriate.

Percentages and absolute counts of the different BALF cell subpopulations were compared among participants with HP, sarcoidosis and other DLD. Additionally, we performed a logistic regression to compare participants with a diagnosis of HP with the remaining patients over the percentage and number of each lymphocyte subpopulation (NK, NKT and CD8^+^ cells) in BALF – after performing an univariable analysis, we performed a multivariable analysis, adjusting the values for each lymphocyte subpopulation for the other subpopulations. We also performed a similar analysis with the diagnosis of sarcoidosis as dependent variable. Associations are presented as odds ratios (ORs) with 95% confidence intervals.

Moreover, we assessed the validity of the percentage and number of each BALF lymphocyte subpopulation in the diagnosis of HP versus the remaining DLD, by assessing the respective area under (AUC) the receiver operating characteristic (ROC) curves. Sensitivity, specificity, and positive and negative predictive values (PPV and NPV, respectively) were calculated for the different NK, NKT and CD8^+^ BALF lymphocytes cut-offs. The Youden index (J = max [sensitivity+specificity-1]) was used to establish the best cut-off for HP diagnosis. A similar analysis was performed aiming to test each BALF lymphocyte subpopulation in the diagnosis of sarcoidosis versus the remaining DLD.

Statistical analyses were performed using the Statistical Package for Social Sciences (SPSS) version 21.0 (SPSS Inc., Chicago, IL, USA). A *p* value of less than 0.05 was considered statistically significant. When performing several pairwise comparisons on the same variable, a Bonferroni correction for the *p*-value was adopted.

## Results

**Fig. 1:**
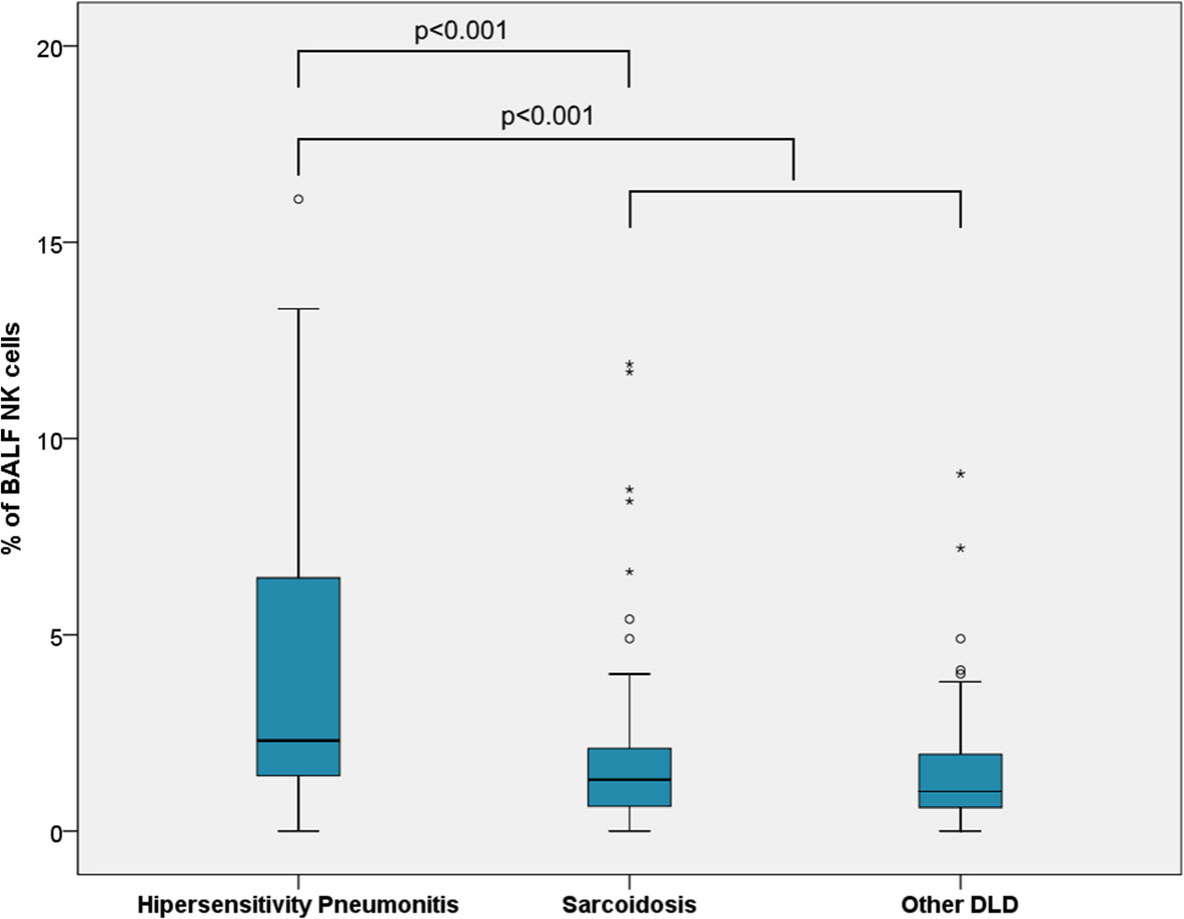
Bronchoalveolar lavage fluid (BALF) NK cells (%) in patients with diffuse lung diseases (DLDs). NK cell levels were significantly higher in patients with hypersensitivity pneumonitis compared with patients with sarcoidosis (*p* < 0.001) and other DLDs (*p* < 0.001). White circles represent mild outliers and stars represent extreme outliers (two extreme outliers in the hypersensitivity pneumonitis group - 42.5 and 22.9% - are not represented)

From the 202 subjects enrolled, most had a final diagnosis of sarcoidosis (*n* = 106; 52.5%), followed by HP (*n* = 53; 26.2%) and other DLD (*n* = 43; 21.3%). In the other DLD group there were 25 patients with CTD-ILD, 10 with OP, and 8 with IPF. The patients had a mean (SD) age of 46.8 (15.8) years; 41.6% were male and 74.8% were non-smokers. The demographic and BALF characteristics of the different groups are shown in Table 1. All the patients except those with sarcoidosis had mixed alveolitis. Median BALF lymphocytosis was 41.8%, and patients with HP had significantly higher lymphocytosis than those with sarcoidosis (53.2% vs 39.9%, respectively; *p* < 0.001) or other DLD (53.2% vs 31.2%; *p* < 0.001). Except for NKT and B cells, we found significant differences regarding the cell percentages of the different BALF lymphocyte subsets across different interstitial lung diseases (Table 2). The percentage of BALF NK cells was significantly higher in patients with HP than in those with sarcoidosis (2.3% vs 1.3%; *p* < 0.001), or other DLD (2.3% vs 1.0%; *p* < 0.001) (Fig. 1). A similar pattern was found for the absolute numbers of BALF NK cells (Additional file 2), for which the median count was higher in the HP group than for in the other DLD group (2453 vs 532 cells/mL; *p* < 0.001).

**Table 1:** Demographics and bronchoalveolar lavage fluid characteristics of patients with diffuse lung diseases (DLD) by diagnosis

	All patients (*n* = 202)	Sarcoidosis (*n* = 106)	Hypersensitivity pneumonitis (*n* = 53)	Other DLDs			
				All (*n* = 43)	Organising pneumonia (*n* = 10)	Pulmonary involvement in connective tissue diseases (*n* = 25)	Idiopathic pulmonary fibrosis (*n* = 8)
Age, years (mean ± SD)^*^	46.8 ± 15.8	38.7 ± 11.2	51.3 ± 16.3	61.5 ± 12.0	57.9 ± 11.4	59.1 ± 11.3	73.4 ± 8.2
Gender (female/male)^†^	118/84	60/46	32/21	26/17	5/5	20/5	1/7
Smoking status (no/yes)^†† a^	151/48	78/26	44/9	29/13	7/2	20/5	2/6
Total cell count ^b^, ×10^5^cells/mL (median, P25-P75)^**^	1.7 (0.9–3.3)	1.6 (0.8–2.8)	2.8 (1.2–6.1)	1.4 (0.8–2.4)	1.6 (0.7–2.4)	1.2 (0.8–2.5)	1.5 (1.0–3.6)
Neutrophils ^b^,% (median, P25-P75)^*^	2.8 (1.2–5.8)	1.6 (1.0–3.0)	4.8 (2.7–8.2)	5.8 (2.4–9.8)	8.7 (5.0–9.9)	3.6 (1.5–9.8)	7.2 (4.7–16.5)
Eosinophils ^b^, % (median, P25-P75)^*^	0.6 (0.2–2.0)	0.4 (0.1–1.0)	1.6 (0.5–3.7)	0.8 (0.2–2.8)	1.6 (0.4–6.5)	0.4 (0.0–1.5)	2.5 (1.1–8.7)
Alveolar macrophages ^b^, % (median, P25-P75)^*^	49.2 (34.6–65.2)	56.0 (37.6–68.7)	32.4 (22.9–49.0)	55.2 (42.0–66.4)	44.1 (28.1–58.2)	55.8 (43.3–68.2)	58.6 (44.7–65.3)
Lymphocytes ^b^, % (median, P25-P75)^*^	41.8 (28.0–57.4)	39.9 (23.9–56.6)	53.2 (41.0–69.1)	31.2 (23.8–43.8)	42.3 (27.6–57.1)	30.0 (23.7–44.8)	26.8 (20.1–33.0)

*SD* standard-deviation, *P* percentile

^*^p < 0.001 for the comparison between sarcoidosis, hypersensitivity pneumonitis and all other DLD (Kruskal-Wallis test); ^†^*p* = 0.302 for the comparison between sarcoidosis, hypersensitivity pneumonitis and all other DLD (chi-square test); ^††^*p* = 0.274 for the comparison between sarcoidosis, hypersensitivity pneumonitis and all other DLD (chi-square test); ^**^p = 0.001 for comparison between sarcoidosis, hypersensitivity pneumonitis and all other DLD (Kruskal-Wallis test)

^a^No information for 3 patients – 2 with sarcoidosis and 1 with organizing pneumonia

^b^Numbers obtained from microscopic counting

**Table 2:** Percentages of lymphocyte subsets in bronchoalveolar lavage fluid of patients with diffuse lung diseases (DLD)

Median percentage (P25-P75)	All patients (*n* = 202)	Sarcoidosis (*n* = 106)	Hypersensitivity pneumonitis (*n* = 53)	Other DLDs			
				All (*n* = 43)	Organising pneumonia (*n* = 10)	Pulmonary involvement in connective tissue diseases (*n* = 25)	Idiopathic pulmonary fibrosis (*n* = 8)
T lymphocytes^*^	92.5 (87.5–95.9)	94.1 (90.0–96.4)	88.1 (83.0–92.9)	93.5 (87.2–96.6)	93.1 (86.0–95.4)	93.7 (88.8–96.7)	93.7 (87.4–96.9)
CD4^+^ T cells^*^	69.5 (46.8–82.3)	77.8 (67.5–84.7)	54.0 (36.0–73.8)	51.6 (29.3–71.9)	45.7 (30.7–68.8)	55.0 (38.3–72.1)	37.1 (25.0–80.2)
CD8^+^ T cells^*^	24.9 (13.2–48.4)	18.1 (11.9–28.6)	39.1 (21.2–57.8)	46.8 (24.8–65.7)	50.4 (24.9–64.8)	40.8 (24.2–58.3)	56.8 (18.2–72.7)
CD4/CD8 ratio^*^	2.8 (1.0–6.3)	4.3 (2.4–7.1)	1.4 (0.6–3.5)	1.1 (0.5–2.9)	0.9 (0.5–2.8)	1.4 (0.7–3.0)	0.7 (0.3–5.2)
NK cells^*^	1.5 (0.8–2.6)	1.3 (0.6–2.1)	2.3 (1.4–6.5)	1.0 (0.5–2.0)	0.9 (0.3–1.3)	1.1 (0.6–2.1)	1.2 (0.7–4.1)
NKT cells^†^	2.9 (1.3–6.4)	2.8 (1.4–5.6)	3.8 (1.7–8.2)	2.7 (1.1–6.4)	2.9 (1.2–8.5)	2.6 (1.0–6.5)	2.2 (0.8–5.3)
B lymphocytes^**^	0.3 (0.1–0.8)	0.3 (0.1–0.7)	0.6 (0.2–1.1)	0.4 (0.1–1.1)	0.4 (0.2–0.8)	1.0 (0.2–1.6)	0.2 (0.1–0.5)

*P* percentile

^*^p < 0.001 for the comparison between sarcoidosis, hypersensitivity pneumonitis, and all other DLDs (Kruskal-Wallis test); ^†^*p* = 0.188 for the comparison between sarcoidosis, hypersensitivity pneumonitis, and all other DLDs (Kruskal-Wallis test); ^**^p = 0.266 for the comparison between sarcoidosis, hypersensitivity pneumonitis, and all other DLDs (Kruskal-Wallis test)

In the HP group, the median absolute number of BALF NK cells was significantly higher in patients with a restrictive pattern in lung function tests (*n* = 23; 6687 cells/mL) than in those with an obstructive pattern (*n* = 13; 903 cells/mL) or normal lung function (*n* = 13; 1683 cells/mL) (*p* < 0.001). We also observed a trend for higher BALF NK cells absolute numbers in HP patients with an acute/subacute (*n* = 16) versus a chronic (*n* = 27) clinical presentation (9520 vs 1962 cells/mL, respectively; *p* = 0.059).

On the other hand, in patients with sarcoidosis, the median absolute number of BALF NK cells was not significantly different between patients with a restrictive pattern (*n* = 17; 688 cells/mL), an obstructive pattern (*n* = 25; 613 cells/mL) or normal lung function (*n* = 48; 836 cells/mL) (*p* = 0.676). Accordingly, no significant differences were observed when comparing the median absolute number of BALF NK cells across different radiologic stages of sarcoidosis – stage I (*n* = 33; 688.2 cells/mL); stage II (*n =* 58; 768.4 cells/mL); stage III/IV (*n* = 7; 836.2 cells/mL) (*p* = 0.941) –, as well as when comparing patients in which sarcoidosis resolved (*n* = 38; 728.9 cells/mL) versus those in which sarcoidosis evolved to a chronic condition (*n* = 40; 935.5 cells/mL) (*p* = 0.873). Similar results are obtained when assessing the percentage of NK cells in BALF. No significant differences were observed when comparing the absolute numbers or the percentages of NKT cells in sarcoidosis patients regarding the disease pattern, radiologic stage or evolution.

In the logistic regression, HP diagnosis was established as the dependent variable, while percentages of BALF NK, NKT and CD8^+^ cells were tested as independent variables. After adjusting for the percentage of NKT and CD8^+^ cells, the percentage of NK cells was significantly associated with a higher probability of having a diagnosis of HP (OR = 1.29 *per* each 1% increase in the percentage of NK cells; 95%CI = 1.14–1.45; *p* < 0.001). The CD8^+^ cell percentage was also significantly associated with an HP diagnosis, even after adjustment for NK and NKT cells (OR = 1.03 *per* each 1% increase in the percentage of CD8^+^ cells; 95%CI = 1.01–1.04; *p* = 0.003) (Table 3A*)*. Similar results were obtained when absolute BALF NK, NKT and CD8^+^ cell counts were used as independent variables (Table 3A). We obtained similar results when restricting the comparison to HP versus sarcoidosis (Table 3B).

**Table 3:** Results of univariate and multivariate analyses with hypersensitivity pneumonitis versus other lung diseases

Lymphocyte populations	Crude OR (95%CI)	*P* value	Adjusted OR (95%CI)	*P* value
A. Hypersensitivity pneumonitis versus other diffuse lung diseases (sarcoidosis and other diffuse lung diseases)				
CD8^+^ (%)	1.02 (1.01–1.04)	0.004	1.03 (1.01–1.04)^a^	0.003
NK (%)	1.28 (1.14–1.45)	< 0.001	1.29 (1.14–1.46)^b^	< 0.001
NKT (%)	1.03 (0.99–1.06)	0.184	1.02 (0.98–1.06)^c^	0.386
CD8^+^ (absolute count)^g^	1.02 (1.01–1.03)	< 0.001	1.01 (1.00–1.02)^d^	0.070
NK (absolute count)^g^	1.25 (1.12–1.39)	< 0.001	1.15 (1.03–1.29)^e^	< 0.001
NKT (absolute count)^g^	1.08 (1.03–1.13)	0.002	1.02 (0.99–1.06)^f^	0.241
B. Hypersensitivity pneumonitis versus sarcoidosis				
CD8^+^ (%)	1.04 (1.03–1.06)	< 0.001	1.05 (1.03–1.07)^a^	< 0.001
NK (%)	1.27 (1.11–1.45)	< 0.001	1.29 (1.12–1.49)^b^	< 0.001
NKT (%)	1.02 (0.98–1.06)	0.282	1.01 (0.97–1.05)^c^	0.725
CD8^+^ (absolute count)^g^	1.02 (1.01–1.03)	< 0.001	1.02 (1.00–1.03)^d^	0.025
NK (absolute count)^g^	1.21 (1.09–1.35)	< 0.001	1.12 (1.00–1.25)^e^	0.055
NKT (absolute count)^g^	1.07 (1.02–1.12)	0.007	1.02 (0.98–1.05)^f^	0.410
C. Sarcoidosis versus all other diffuse lung diseases (hypersensitivity pneumonitis and other diffuse lung diseases)				
CD8^+^ (%)	0.95 (0.94–0.97)	< 0.001	0.95 (0.93–0.97)^a^	< 0.001
NK (%)	0.87 (0.78–0.97)	0.014	0.85 (0.75–0.96)^b^	0.010
NKT (%)	0.99 (0.96–1.03)	0.574	1.00 (0.96–1.04)^c^	0.938
CD8^+^ (absolute count)^g^	0.98 (0.97–0.99)	< 0.001	0.98 (0.97–0.99)^d^	0.005
NK (absolute count)^g^	0.90 (0.83–0.97)	0.007	0.96 (0.90–1.03)^e^	0.239
NKT (absolute count)^g^	0.97 (0.93–1.00)	0.041	0.99 (0.97–1.03)^f^	0.993

Results of univariate and multivariate analyses with hypersensitivity pneumonitis (versus the other diffuse lung diseases – 3A; and versus sarcoidosis only – 3B) and sarcoidosis (versus the other diffuse lung diseases – 3C) as dependent variables. Absolute numbers and percentages of CD8^+^, NK, and NKT cells in the bronchoalveolar lavage fluid were assessed as independent variables

*CI* confidence interval, *NK* natural killer, *OR* odds ratio

^a^adjusted for percentage of NK and NKT cells

^b^adjusted for percentage of CD8+ and NKT cells

^c^adjusted for percentage of CD8+ and NK cells

^d^adjusted for absolute number of NK and NKT cells

^e^adjusted for absolute number of CD8+ and NKT cells

^f^adjusted for absolute number of CD8+ and NK cells

^g^Odds ratio and confidence intervals expressed in number of cells/μL

On the other hand, when sarcoidosis was established as the dependent variable, the percentages of BALF NK and CD8^+^ cells were significantly associated with a lower risk of having a diagnosis of sarcoidosis: adjusted OR = 0.851 *per* each 1% increase in the percentage of NK cells (95%CI = 0.753–0.963; *p* = 0.010); and adjusted OR = 0.949 *per* each 1% increase in the percentage of CD8^+^ cells (95%CI = 0.932–0.966; *p* < 0.001) (Table 3C*)*. Similar results were obtained when assessing the absolute number of CD8^+^ cells.

**Fig. 2:**
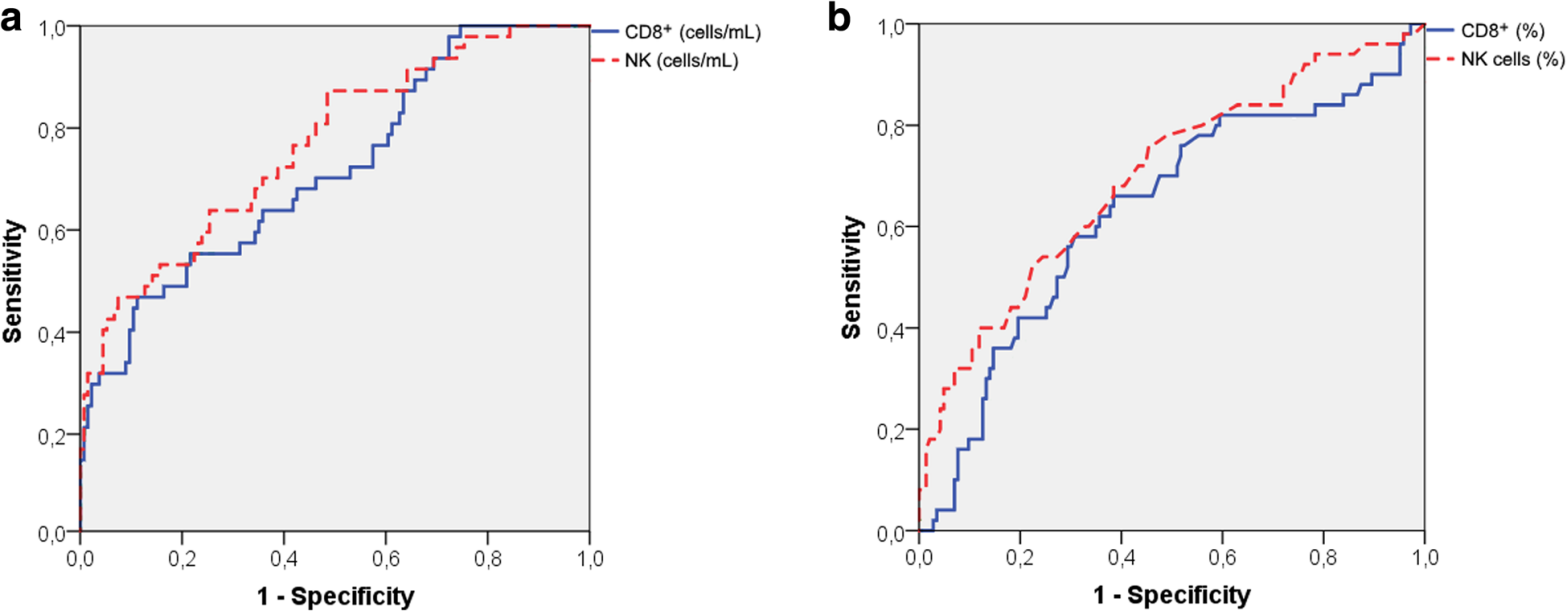
Diagnostic performance of bronchoalveolar lavage fluid NK and CD8^+^ cells in diagnosing hypersensitivity pneumonitis. Receiver operating characteristic (ROC) curves for absolute numbers (**a**) and percentages (**b**) of bronchoalveolar lavage fluid NK and CD8^+^ cells in the diagnosis of hypersensitivity pneumonitis (*n* = 53) versus all other diffuse lung diseases (sarcoidosis + other diffuse lung diseases) (*n* = 149)

We evaluated the performance of selected BALF cytotoxic immunophenotypes in the diagnosis of HP, obtaining the respective ROC curves (Fig. 2 and Table 4). The number of NK cells/mL in the BALF presented the best diagnosis performance of selected immunophenotypes (AUC = 0.76; 95%CI = 0.68–0.84), followed by the number of CD8^+^ cells/mL (AUC = 0.71; 95%CI = 0.63–0.79) (Fig. 2a). For a cut-off of 2000 NK cells/mL in the BALF, the specificity in the diagnosis of HP was of 80%, while the sensitivity was of 49%, with a corresponding PPV of 46% and a NPV of 82%. Alternatively, and using percent values, a cut-off of 2.4% NK cells in the BALF also corresponded to a specificity of 80%, although with a sensitivity of 47%, PPV of 44% and NPV of 81% (Table 5). The maximum Youden index was 1.4% for the NK cell percentage (sensitivity = 77%; specificity = 55%) and 3446 cells/mL for the absolute cell count (sensitivity = 47%; specificity = 92%). The corresponding values for CD8^+^ cells were 28.7% (sensitivity = 65%; specificity = 61%) and 40,671 cells/mL (sensitivity = 46%; specificity = 88%) (Table 4).

**Table 4:** Diagnostic performance of bronchoalveolar lavage fluid NK, NKT and CD8^+^ cells in diagnosing hypersensitivity pneumonitis

Lymphocyte populations	AUC-ROC (95%CI)	Maximum Youden’s index cut-off	Sensitivity - % (95%CI)	Specificity - % (95%CI)	PPV - % (95%CI)	NPV - % (95%CI)
NK (cells/mL)	0.76 (0.69–0.84)	3446	47 (40–54)	92 (88–96)	68 (62–74)	83 (78–88)
NK (%)	0.70 (0.62–0.79)	1.4	77 (71–83)	55 (48–62)	37 (30–44)	85 (80–90)
NKT (cells/mL)	0.73 (0.65–0.82)	1678	75 (69–81)	65 (58–72)	42 (35–49)	88 (84–93)
NKT (%)	0.58 (0.49–0.68)	6.1	40 (33–47)	88 (84–93)	39 (33–46)	79 (73–85)
CD8^+^ (cells/mL)	0.71 (0.62–0.80)	40,671	47 (40–54)	88 (84–93)	59 (52–66)	82 (77–87)
CD8^+^ (%)	0.63 (0.54–0.72)	28.7	65 (58–72)	61 (54–68)	39 (33–46)	84 (79–89)

Areas Under the Receiver Operating Characteristic curves (AUC-ROC) for absolute and relative numbers of NK, NKT and CD8^+^ cells in the diagnosis of hypersensitivity pneumonitis (*n* = 53) versus other diffuse lung diseases (sarcoidosis + other diffuse lung diseases) (*n* = 149). Sensitivities, specificities and predictive values are presented for the cut-off points with the maximum Youden’s indices

*CI* confidence interval, *PPV* positive predictive value, *NPV* negative predictive value

**Table 5:** Diagnosis of hypersensitivity pneumonitis versus other diffuse lung diseases (sarcoidosis + other diffuse lung diseases)

Percentage of natural killer cells			Percentage of CD8^+^ cells		
Cut-off	Sn - % (95%CI)	Sp - % (95%CI)	Cut-off	Sn - % (95%CI)	Sp - % (95%CI)
0.5	94 (91–97)	21 (15–27)	15	83 (78–88)	32 (26–38)
1.0	84 (79–89)	37 (30–44)	20	78 (72–84)	45 (38–52)
1.5	73 (67–79)	57 (50–64)	25	65 (58–72)	56 (49–63)
2.0	55 (48–62)	73 (67–79)	30	61 (54–68)	63 (56–70)
2.5	45 (38–52)	81 (76–86)	35	57 (50–64)	67 (61–74)
3.0	41 (34–48)	86 (81–91)	40	49 (42–56)	72 (66–78)
3.5	37 (30–44)	88 (84–92)	45	41 (34–48)	77 (71–83)
4.0	33 (27–40)	92 (88–96)	50	35 (28–42)	81 (76–86)
4.5	31 (25–37)	93 (89–97)	55	29 (23–35)	86 (81–91)
5.0	27 (21–33)	93 (89–97)	60	18 (13–23)	87 (82–92)
Absolute number of natural killer cells/mL			Absolute number of CD8^+^ cells/mL		
Cut-off	Sn - % (95%CI)	Sp - % (95%CI)	Cut-off	Sn - % (95%CI)	Sp - % (95%CI)
500	88 (84–92)	50 (43–57)	15,000	61 (54–68)	63 (56–70)
1000	65 (58–72)	65 (58–72)	20,000	55 (48–62)	73 (67–79)
1500	59 (52–66)	74 (68–80)	25,000	53 (46–60)	78 (72–84)
2000	53 (46–60)	78 (72–84)	30,000	49 (42–56)	79 (73–85)
2500	49 (42–56)	86 (81–91)	35,000	47 (40–54)	84 (79–89)
3000	47 (40–54)	91 (87–95)	40,000	47 (40–54)	87 (82–92)
3500	47 (40–54)	92 (88–96)	45,000	45 (38–52)	89 (85–93)
4000	43 (36–50)	94 (91–97)	50,000	41 (34–48)	89 (85–93)
4500	43 (36–50)	94 (91–97)	55,000	33 (27–40)	91 (87–95)
5000	41 (34–48)	95 (92–98)	60,000	33 (27–40)	93 (89–97)

Sensitivities and specificities for different cut-offs for absolute numbers and percentages of CD8^+^ and natural killer cells in bronchoalveolar lavage fluid

*Sn* Sensitivity, *Sp* Specificity

We performed similar analyses regarding the diagnosis of sarcoidosis, but only the BALF percentage and the absolute number of CD8^+^ showed an acceptable diagnostic performance (i.e., an AUC-ROC significantly different from 0.5). For the BALF percentage of CD8^+^ cells, the AUC was of 0.76 for ruling out sarcoidosis (95%CI = 0.69–0.82), with the maximum Youden index obtained with 28.7% (sensitivity = 0.68; specificity = 0.76). For the BALF absolute number of CD8^+^ cells, the AUC was of 0.68 for ruling out sarcoidosis (95%CI = 0.60–0.75), with the maximum Youden index obtained with 38,070 cells/mL (sensitivity = 92%; specificity = 31%).

Analysis of peripheral blood showed no significant differences regarding the median percentages of NK cells (sarcoidosis: 12.9%; HP: 14.9%; other DLD: 12.4%; *p* = 0.758), NKT cells (sarcoidosis: 4.0%; HP: 3.4%; other DLD: 2.6%; *p* = 0.088) and CD8^+^ cells (sarcoidosis: 29.8%; HP: 36.5%; other DLD: 28.0%; *p* = 0.297) between the different DLD. Nevertheless, when adjusted for the other peripheral blood cell populations, the percentage of CD8^+^ was significantly associated with higher chances of having HP (OR = 1.03 *per* 1% increase; 95%CI = 1.00–1.05; *p* = 0.025), while the percentage of NKT cells was significantly associated with higher chances of having sarcoidosis (OR = 1.07 *per* 1% increase; 95%CI = 1.01–1.13; *p* = 0.032). No significant association was found regarding the percentage of any peripheral blood cell population with the pattern of HP, or with the pattern, radiologic stage or evolution of sarcoidosis (data not shown).

## Discussion

In this study, absolute and relative BALF NK cell counts were independently and significantly associated with a diagnosis of Hypersensitivity Pneumonitis in a series of patients with DLD and lymphocytic alveolitis. Moreover, absolute counts were associated with restrictive lung function impairment and an acute/subacute clinical presentation. We prospectively enrolled a diverse range of patients with DLD, as BALF profiling is a useful differential diagnostic aid in daily clinical practice [3, 30]. With such a wide spectrum of entities, a combination of lymphocytic alveolitis and elevated NK cell counts may help to discriminate patients with HP from those with other DLD.

Nevertheless, while information on the NK cell counts might be useful on the diagnostic classification of DLD, further research may be needed on the practical role of such counts within a multidisciplinary approach of a patient with DLD; in this instance, future studies should assess the impact of taking into account the number of BALF NK cells in the differential diagnosis of DLD. This is particularly relevant, as some of the differences found (e.g., regarding the percentage of BALF NK cells across different DLD entities) – although significant – do not have a large magnitude, possibly limiting its clinical use. Another important limitation of this study is that our results may only be generalizable to patients with lymphocytic alveolitis (BALF lymphocytes > 15%). Although we investigated the most clinically common DLDs, there was a large proportion of patients with HP in our series due to high levels of exposure to avian proteins (bird fanciers) and molds (indoor home exposure and occupational exposure in the cork industry) in northern Portugal [28, 31]. Most included HP patients had subacute or chronic presentations, as acute presentations are rare in our clinical practice; while a high BALF lymphocytosis is less frequently observed in chronic presentations, these forms - especially with interstitial pneumonias as a usual interstitial pneumonia (UIP) ‘like’ patterns - are the most frequent in our setting. On the other hand, the unexpectedly low frequency of patients with OP in our series results from the fact that, in our DLD center, patients with OP are usually diagnosed through a Computer Tomography-guided transthoracic biopsy and do not perform BALF. Also, in patients with CTD-ILD, BALF is only performed when that are any particular issues related with the differential diagnosis of lung involvement, as this condition is usually diagnosed according to the autoimmune background and characteristic radiological features on HRCT-scan. The eight patients with IPF in our series belong to the minority (approximately 20%) of patients with IPF and increased lymphocytes in BALF. Mild neutrophilia and mild eosinophilia, by contrast, are common BALF findings [20, 32]. Nevertheless, all the patients with IPF were diagnosed according to the 2011 ERS/ATS criteria, which include (i) absence of any environmental exposure suggesting other diagnoses, and (ii) a negative autoimmune panel test associated with a HRCT scan with definitive UIP [20]. In cases of inconsistency (e.g., signs suggestive of UIP on the HRCT scan) and in patients with an adequate clinical condition, a surgical lung biopsy was performed. In any case, all the diagnoses were discussed in a multidisciplinary team setting, blinded to the results of the cytotoxic lymphocyte subsets phenotyped [33, 34].

Although international consensus statements have been published on the classification of idiopathic interstitial pneumonia, standardization of diagnostic guidelines is still lacking for some DLD, such as HP [18, 22]. A number of diagnostic criteria and prediction rules have been proposed for HP, but their diagnostic accuracy has not been sufficiently validated [35–37]. While a higher frequency of NK cells has been reported in HP patients, the diagnostic value of this finding in BALF had not yet been established [6, 7]. To our knowledge, our study is the first to indicate absolute and relative BALF NK cell count cut-offs (2000/mL and 2.4%) with a specificity and NPV of over 80% for the diagnosis of HP.

Concerning NKT cells, our results contrast with previous studies assessing its frequency in BALF [9, 38]. In fact, Tondell et al., found that the association of high values of NKT cells and activated CD8^+^ cells was more suggestive of HP than sarcoidosis, while Korosec et al. reported a greater fraction of BALF NKT cells (mostly CD8^+^ lymphocytes) in HP than in sarcoidosis [9, 38]. Both of these studies, however, assessed few HP patients (*n* = 10 and *n* = 17, respectively) and only performed a comparative analysis with sarcoidosis.

To our knowledge, the present study is also the first to describe a higher number of NK cells in the BALF of HP patients with acute/subacute forms and with restrictive lung function impairment, further suggesting a role for lung NK cells in the pathogenesis of HP. In fact, while Korosec et al. found no statistically significant differences between HP clinical presentations, only 5 of 17 HP cases were chronic forms [38]. Also, in the study of Papakosta et al., while no differences were found in BALF NK cells between HP acute and subacute forms, none of the 19 studied patients had a chronic presentation [14].

Conventional human NK cells (CD3^−^CD16/56^+^) belong to one of the three innate lymphocyte cell families (group 1 ILCs [ILC1s]) that have been increasingly recognized as key players of immune regulation and human pathology (reviewed in [39]). ILCs, which lack antigen-specific T-cell receptors (TCRs), respond to epithelial- or stromal-derived stress signals by producing an array of cytokines profiles that regulate subsequent immune responses. Both conventional NK cells and other ILC1s mainly produce IFN-gamma on activation, with NK cells exhibiting cytotoxic activity mediated by granzymes and perforin in a similar way as CD8^+^ cytotoxic T cells [40]. In this sense, ILC1s and conventional NK are now considered innate counterparts to type 1 helper (Th1) and cytotoxic T cells that mount a “Th1 / cytotoxic type” immune response independently of antigen recognition [41]. Accordingly, they act as a first line of defense against pathogens and modulate acute and chronic inflammatory disorders in the human lung.

It was also recently shown that human lung-resident NK cells (about 15% of lung leukocytes) are composed of highly differentiated but hypofunctional CD56^dim^/CD16^+^ cells (although typically expressing high levels of perforin), possibly circulating between the blood and lung [42]. These findings suggest that, being lung NK cells mainly non-resident lymphocytes, inflammatory pulmonary disorders may be accompanied by differentiation and functional changes of lung NK cells, putatively overcoming the usual suppressive environment of the normal alveolar space [43]. Our results support the hypothesis that pulmonary NK cells play a particular role in the pathogenesis or in the inflammatory response of DLD, namely as we found that, in patients with different DLD, these cell populations have a significantly different frequency in the BALF but not in the peripheral blood. This view is also in line with the results of several experimental models that have shown that the interstitial lung inflammation dynamics seen in HP (resolution or massive fibrosis) are critically influenced and regulated by NK cell activity and their secreted cytokine profiles [44–46].

## Conclusions

BALF analysis and immunophenotyping of lung NK cells, with widely available and common technics and reagents, seems particularly helpful in cases of DLD with lymphocytic alveolitis. Our results show that a cut-off of 2.4% for the percentage of CD3^−^ 16/56^+^ cells in BALF associates with a diagnosis of HP with a specificity of over 80%. While the functional and immunopathological implications of BALF NK cells deserve further research, our results have practical implications, as they indicate that NK immunophenotyping may be a helpful adjunct to the diagnostic workup of DLD, especially in suspected cases of HP where standardized diagnostic criteria are still lacking.

## Additional files

Additional file 1: **Figure S1.** Flow cytometry analysis of BALF samples. For the gating strategy, lymphocytes were distinguished on the basis of forward (FSC) versus side (SSC) scatters (A) and additional gating was applied using SSC versus CD45 (B). T cells were gated by their expression of CD3 (C), and NK and NKT-like cells by CD3 versus CD16/CD56 expression (D). (TIF 123 kb)

Additional file 2: **Table S1.** Absolute numbers of lymphocyte subsets in bronchoalveolar lavage fluid and CD4/CD8 ratios of 202 patients with diffuse lung disease (DLD) by diagnosis. (DOCX 15 kb)

## Abbreviations

AUC: Area under curve
BALF: Bronchoalveolar lavage fluid
CTD-ILD: Connective tissue disease with pulmonary involvement
DLD: Diffuse lung diseases
ERS/ATS: European Respiratory Society/American Thoracic Society
HP: Hypersensitivity pneumonitis
HRCT: High-resolution computed tomography
IPF: Idiopathic pulmonary fibrosis
NK: Natural killer
NKT: Natural killer T cells
OP: Organizing pneumonia
PPV and NPV: Positive and negative predictive values
ROC: Receiver operating characteristic curve
