# Supplementary figures and images for: NK and NKT cells in the diagnosis of diffuse lung diseases presenting with a lymphocytic alveolitis
Source: BMC Pulm Med. 2019 Feb 13;19:39. doi: 10.1186/s12890-019-0802-1 (PMC6373142; doi:10.1186/s12890-019-0802-1)

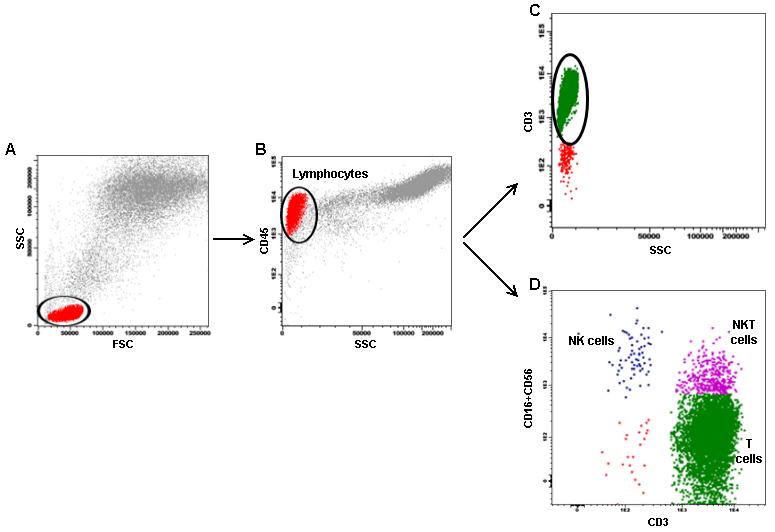

Supplement: Supplementary file 1 — Figure S1. Flow cytometry analysis of BALF samples. For the gating strategy, lymphocytes were distinguished on the basis of forward (FSC) versus side (SSC) scatters (A) and additional gating was applied using SSC versus CD45 (B). T cells were gated by their expression of CD3 (C), and NK and NKT-like cells by CD3 versus CD16/CD56 expression (D). (TIF 123 kb) [file 12890_2019_802_MOESM1_ESM.tif]
