# Supplementary material for: NK and NKT cells in the diagnosis of diffuse lung diseases presenting with a lymphocytic alveolitis
Source: BMC Pulm Med. 2019 Feb 13;19:39. doi: 10.1186/s12890-019-0802-1 (PMC6373142; doi:10.1186/s12890-019-0802-1)
Supplement: Supplementary file 2 — Table S1. Absolute numbers of lymphocyte subsets in bronchoalveolar lavage fluid and CD4/CD8 ratios of 202 patients with diffuse lung disease (DLD) by diagnosis. (DOCX 15 kb) [file 12890_2019_802_MOESM2_ESM.docx]

**Additional file 2** Absolute numbers of lymphocyte subsets in bronchoalveolar lavage fluid and CD4/CD8 ratios of 202 patients with diffuse lung disease (DLD) by diagnosis.

| Median absolute number of cells/mL (P25-P75) | All patients  (n=202) | Sarcoidosis (n=106) | Hypersensitivity pneumonitis (n=53) | Other DLDs | | | |
| --- | --- | --- | --- | --- | --- | --- | --- |
|  |  |  |  | All  (n=43) | Organising pneumonia (n=10) | Pulmonary involvement in connective tissue diseases (n=25) | Idiopathic pulmonary fibrosis (n=8) |
| T lymphocytes* | 48,571 (19,009-109,719) | 38,184 (16,620-91,867) | 81,347 (43,194-238,806) | 33,909 (12,927-79,058) | 48,889 (14,029-109,185) | 34,271 (9,349-80,657) | 24,738 (16,198-66,357) |
| CD4^+^ T cells* | 26,423 (11,466-74,451) | 27,274 (11,570-73,660) | 51,099 (19,909-104,351) | 12,467 (5,748-30,371) | 17,453 (5,730-59,364) | 10,675 (4,613-44,543) | 12,638 (6,626-19,135) |
| CD8^+^ T cells* | 10,478 (4,715-33,899) | 7,486 (3,116-18,741) | 26,511 (6,368-115,340) | 16,491 (4,469-39,912) | 20,584 (7,299-46,278) | 11,208 (3,794-38,016) | 17,600 (2,700-42,506) |
| NK cells* | 803 (295-2,419) | 706 (322-1,964) | 2,453 (727-12,561) | 334 (130-1,009) | 178 (93-962) | 412 (143-1,063) | 285 (171-2,802) |
| NKT cells* | 1,486 (789-4,052) | 1,382 (724-3,203) | 3,539 (1,529-20,384) | 1,129 (433-1,647) | 1,174 (677-3,435) | 1,150 (466-1,518) | 776 (223-2,880) |
| B lymphocytes** | 88 (12-615) | 65 (0-527) | 157 (0-1,586) | 80 (27-403) | 168 (37-414) | 99 (43-484) | 37 (4-315) |

P=percentile; * *p*<0.001 for the comparison between sarcoidosis, hypersensitivity pneumonitis, and all other DLDs (Kruskal-Wallis test); ** *p*=0.243 for the comparison between sarcoidosis, hypersensitivity pneumonitis, and all other DLDs (Kruskal-Wallis test).
